# Injectable Biodegradable Chitosan–PEG/PEG–Dialdehyde Hydrogel for Stem Cell Delivery and Cartilage Regeneration

**DOI:** 10.3390/gels10080508

**Published:** 2024-08-01

**Authors:** Xiaojie Lin, Ruofan Liu, Jacob Beitzel, Yang Zhou, Chloe Lagadon, Miqin Zhang

**Affiliations:** 1 Department of Materials Science and Engineering, University of Washington, Seattle, WA 98195, USA; xjlin@uw.edu (X.L.); rfliu@uw.edu (R.L.); jbeitz2@uw.edu (J.B.); yz0426@uw.edu (Y.Z.); clagadon@uw.edu (C.L.); 2 Department of Neurological Surgery, University of Washington, Seattle, WA 98195, USA

**Keywords:** knee osteoarthritis, injectable hydrogels, natural polysaccharides, synthetic polymers, stem cells, cartilage regeneration

## Abstract

Stem cell-based therapy holds promise for cartilage regeneration in treating knee osteoarthritis (KOA). Injectable hydrogels have been developed to mimic the extracellular matrix (ECM) and facilitate stem cell growth, proliferation, and differentiation. However, these hydrogels face limitations such as poor mechanical strength, inadequate biocompatibility, and suboptimal biodegradability, collectively hindering their effectiveness in cartilage regeneration. This study introduces an injectable, biodegradable, and self-healing hydrogel composed of chitosan–PEG and PEG–dialdehyde for stem cell delivery. This hydrogel can form in situ by blending two polymer solutions through injection at physiological temperature, encapsulating human adipose-derived stem cells (hADSCs) during the gelation process. Featuring a 3D porous structure with large pore size, optimal mechanical properties, biodegradability, easy injectability, and rapid self-healing capability, the hydrogel supports the growth, proliferation, and differentiation of hADSCs. Notably, encapsulated hADSCs form 3D spheroids during proliferation, with their sizes increasing over time alongside hydrogel degradation while maintaining high viability for at least 10 days. Additionally, hADSCs encapsulated in this hydrogel exhibit upregulated expression of chondrogenic differentiation genes and proteins compared to those cultured on 2D surfaces. These characteristics make the chitosan–PEG/PEG–dialdehyde hydrogel–stem cell construct suitable for direct implantation through minimally invasive injection, enhancing stem cell-based therapy for KOA and other cell-based treatments.

## 1. Introduction

Knee osteoarthritis (KOA) is a chronic condition affecting over 9.3 million American adults, characterized by chronic pain and joint degeneration [1,2,3,4,5]. Its complex pathophysiology includes joint inflammation, neovascularization, and nerve growth. Current treatments primarily focus on symptom managements using medication and surgery, rather than stopping disease progression or repairing damaged tissues, largely due to the limited self-healing capabilities of cartilage [6,7,8,9,10,11,12,13,14]. Stem cell therapy offers significant promise for tissue regeneration in KOA [15,16,17,18]. However, current stem cell delivery methods yield low cell viability at the injection site due to cell dispersion and harsh injection procedures [19,20,21].

Hydrogels offer a promising solution to delivering stem cells by containing the cells within their porous structure at target sites [8,22,23]. Synthetic polymers are commonly used to prepare hydrogels due to their adjustable mechanical properties and stability [24]. However, the synthetic polymers have significant limitations: they lack bioactive motifs necessary for cell adhesion, proliferation, and differentiation; they degrade at unpredictable rates, either being too rapid or too slow; and they often do not match the mechanical properties of natural tissues [25,26,27].

In contrast, natural polysaccharides like chitosan overcome these issues. Chitosan contains bioactive motifs that promote cellular interactions, degrades predictably in sync with tissue healing, and has tuned mechanical properties that closely mimic the extracellular matrix, providing a better environment for cell growth and tissue formation [28,29,30,31]. In addition, chitosan bears anti-tumor and immune regulation characteristics that are commonly desired for biomedical applications [32]. Despite its poor water solubility under physiological conditions, chitosan’s solubility in water can be enhanced through the conjugation of hydrophilic chains. Consequently, chitosan-based hydrogels are more promising for tissue engineering than synthetic ones. A comprehensive comparison of various polysaccharide-based hydrogel systems, including their chemical structures, specific gelation factors, properties, and applications for the treatment of knee osteoarthritis, has been reported previously [8].

The injectability of stem cell-laden hydrogels is critical to the non-invasive treatment of KOA as it allows precise delivery and distribution of therapeutic cells within the joint, minimizing surgery and enhancing tissue regeneration. There are two main strategies for preparing injectable hydrogels: (a) in situ gelation, where liquid polymers are injected at low injection force and then gelate at the target site, allowing controlled and localized hydrogel formation within the body; and (b) the use of shear-thinning polymers, which temporarily decrease in viscosity under shear stress during injection, making them easier to administer. Once injected, these polymers regain their original viscosity, forming a stable hydrogel at the desired location [8,33,34,35]. Additionally, self-healing properties are necessary to effectively fill and conform to the defects’ structure, ensuring stable and continuous tissue support. However, achieving easy injectability and rapid self-healing at physiological conditions presents a significant challenge.

**Scheme 1:**
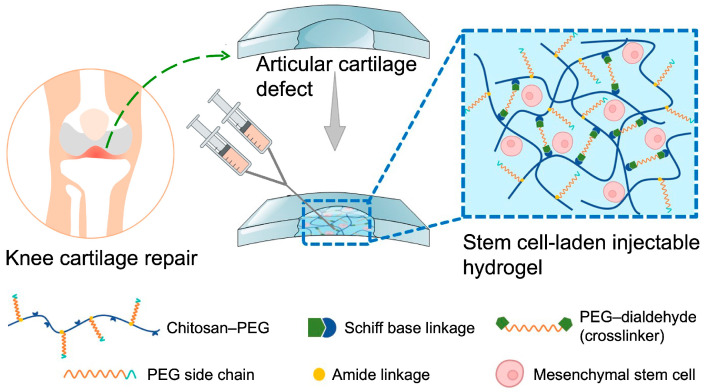
Schematic illustration of an injectable and biological compatible stem cell-laden hydrogel at a cartilage defect site for knee cartilage repair.

In this study, we introduce an injectable, biodegradable, self-healing, and in situ-forming polysaccharide hydrogel, chitosan–PEG/PEG–dialdehyde, to encapsulate and preserve hADSCs for cartilage regeneration (Scheme 1). The primary structure of chitosan comprises a repeating glucosamine unit which is the main component of the cartilage matrix and is an essential ingredient for the synthesis of glycoproteins and glycolipids [36]. The hydrogel was constructed through both a Schiff base reaction and hydrophobic interaction by gently mixing two solutions, one containing chitosan–PEG (modified chitosan) and the other containing PEG–dialdehyde (crosslinker). The Schiff base bond, a dynamic covalent binding, enhances both injectability and self-healing properties by allowing the hydrogel to reform bonds and maintain structural integrity after deformation. Schiff base reactions offer advantages such as mild reaction conditions, precise gelation control, and tunable mechanical properties. PEG is one of the most favorable hydrophilic biocompatible polymers. Thus, incorporating chitosan with modified PEG can (a) enhance the water solubility of chitosan for gelation in water and (b) crosslink chitosan through Schiff base reactions, resulting in a hydrogel with improved properties, including easy injectability and rapid self-healing.

The chitosan–PEG was synthesized through amide linkage between the amino group of chitosan and the carboxyl group of mPEG. The crosslinker PEG–dialdehyde was synthesized by oxidation of terminal hydroxyl groups of PEG 2000. The injectable solutions were prepared individually and subsequently mixed upon injection to the cartilage defects utilizing a double-barrel syringe (Scheme 1). The hADSCs were pre-suspended in the chitosan–PEG solution and encapsulated in situ during the gelation process. The gelation ability, injectability, pore size, self-healing capability, rheological properties, and degradation and swelling behaviors of the formed hydrogels were assessed. Additionally, we examined the temporal changes in the morphology and viability of hADSCs within the encapsulated hydrogels, as well as alterations in gene and protein expression levels associated with chondrogenic differentiation, to inhibit degeneration and promote regeneration of knee cartilage.

## 2. Results and Discussion

### 2.1. Polymer Synthesis and Characterization

Chitosan is generally not water soluble under physiological conditions, often requiring further modification to improve its water solubility. The presence of amino and hydroxyl functional groups on the chitosan backbone renders it conducive to various chemical modifications, such as amidation, esterification, and etherification [8,37,38]. Furthermore, to generate a hydrogel suitable for encapsulating and delivering stem cells for cartilage regeneration, it is essential that all components of the hydrogels must be non-toxic and easily prepared. To meet these criteria, chitosan was modified with mPEG to increase its solubility in a neutral, aqueous, and injectable solution.

**Figure 1:**
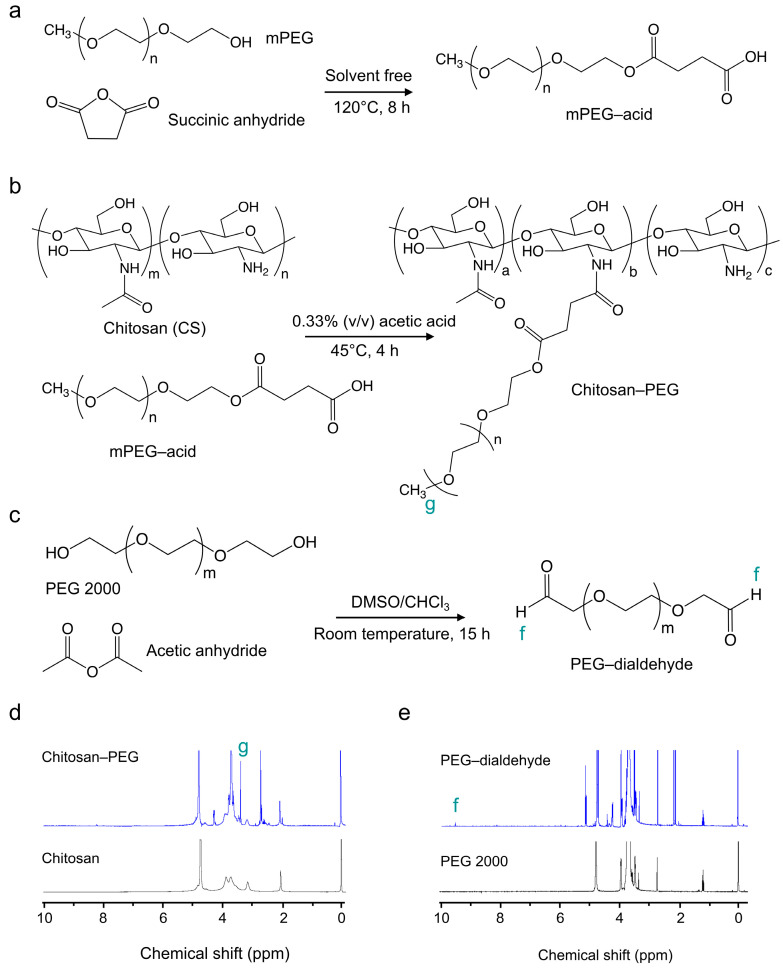
Polymer structure and characterizations. Synthesis routes and chemical structures of (**a**) mPEG–acid, (**b**) chitosan–PEG, and (**c**) PEG–dialdehyde. (**d**) ^1^H NMR spectra of chitosan and chitosan–PEG. (**e**) ^1^H NMR spectra of PEG-2000 and PEG–dialdehyde.

The synthesis route of chitosan–PEG is shown in Figure 1a,b, and the chemical structure was confirmed by ^1^H NMR (Figure 1d). Grafting ratio analysis based on integral values of characteristic peaks revealed that the mPEG grafting ratio in chitosan–PEG is 14%, with 3.25 ppm (1H) representing chitosan and 1.45–1.63 ppm (3H) representing mPEG. By conjugating the mPEG side-chain, the solubility of the modified chitosan in a neutral aqueous solution significantly increased. This modification also resulted in chitosan–PEG becoming a thermosensitive polymer hydrogel that undergoes a sol-to-gel transition in response to temperature changes.

Despite the capacity of chitosan–PEG hydrogels to undergo physical crosslinking via temperature modulation, it was observed that both their storage modulus (G’) and loss modulus (G”) were unacceptably low, approximately ~1 Pa. This deficiency hindered the ability to maintain the 3D encapsulation state for the loaded stem cells [39,40]. To address this issue, additional crosslinking is needed to enhance the mechanical properties of the hydrogel. We used a Schiff base reaction to further crosslink chitosan–PEG through covalent bonding. The Schiff base reactions for preparing polymer hydrogels offer advantages, including its biocompatibility under mild conditions, precise control over gelation, in situ formation, and tunable mechanical properties. A crosslinking agent derived from PEG, PEG–dialdehyde, was synthesized by oxidation of PEG 2000 with DMSO and acetic anhydride (Figure 1c). The DMSO/chloroform mixture ensures complete dissolution of PEG 2000 and PEG–dialdehyde, facilitating efficient oxidation and purification through precipitation in diethyl ether. PEG was chosen as the backbone for providing aldehyde groups due to its excellent biocompatibility, chemical stability, and flexibility in functionalization. PEG allows for precise control over molecular weight and functional group density, which are critical for optimizing the hydrogel’s mechanical strength, swelling behavior, and interaction with encapsulated cells. The Schiff base reaction is anticipated to take place between the amino group of chitosan–PEG and the aldehyde group of PEG–dialdehyde. The production of PEG–dialdehyde was confirmed through ^1^H NMR analysis (Figure 1e), with a characteristic peak at 9.59 ppm, signifying the successful introduction of aldehyde groups to the PEG chain. The yield of PEG–dialdehyde synthesis was found to be 78%.

### 2.2. Hydrogel Preparation and Characterization

#### 2.2.1. Injectability, Morphology, and Self-Healing of Hydrogels

**Figure 2:**
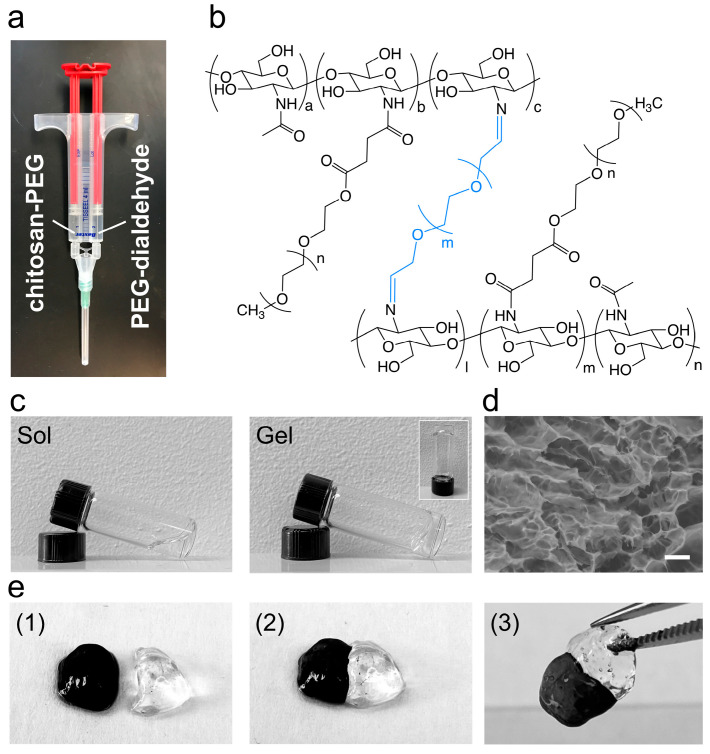
Hydrogel injectability, chemical structure, gelation, morphologies, and self-healing capability. (**a**) Illustration of the double-barrel syringe filled with chitosan–PEG and PEG–dialdehyde solutions for creating hydrogels. (**b**) Chemical structure of crosslinked chitosan–PEG/PEG–dialdehyde hydrogel. (**c**) Gelation process: photos of chitosan–PEG before (sol) and after (gel) adding PEG–dialdehyde. (**d**) SEM photograph of freeze-dried chitosan–PEG/PEG–dialdehyde hydrogel; scale bar represents 20 μm. (**e**) Images demonstrating the self-healing capability of chitosan–PEG/PEG–dialdehyde hydrogel: (1) A 700 μL aliquot of freshly prepared hydrogel was divided into two pieces with one of them stained with Trypan Blue for identification purposes; (2) following this, the two pieces were brought together at room temperature for 3 min; (3) resulting in the formation of a single hydrogel piece through spontaneous fusion, which was then picked up using tweezers.

The hydrogels developed in this study demonstrated injectability through the following methods: First, the hydrogel was prepared by gently blending chitosan–PEG (2.5%, *w*/*v*) and PEG–dialdehyde (1.0 g/mL) at a volume ratio of 6:1, using a double-barrel syringe (Duploject syringe, Baxter) with a 21 G needle (Figure 2a). The gelation occurred within 3 min in Dulbecco’s phosphate-buffered saline (DPBS) or cell culture medium post-injection, and the mechanism of gelation was attributed to the Schiff base reaction between the amino group of chitosan–PEG and the aldehyde group of PEG–dialdehyde (Figure 2b,c). Thus, the chitosan–PEG/PEG–dialdehyde hydrogels demonstrate excellent injectability, enabling in situ gelation for minimally invasive applications. Additionally, the presence of a Schiff base, which is well-known as a dynamic chemical bond, within the hydrogel network can impart shear-thinning and self-healing properties to the material, allowing the hydrogel to flow through narrow-gauge needles or catheters without significant resistance.

As shown in Figure S1a, the viscosity of the chitosan–PEG/PEG–dialdehyde hydrogel dramatically decreased with an increase in the shear rate, indicating that a shear-thinning hydrogel with non-Newtonian properties was created. Thus, this hydrogel is injectable for delivering therapeutic agents. Once the shear stress is removed, the reversible chemical bonds can reassemble, restoring the gel-like state and the mechanical integrity of the hydrogel (Figure S1b). This behavior is critical for injectable hydrogels as it enables them to be easily administered through minimally invasive techniques while retaining their ability to form stable gels at target sites.

Previous research has established that pore sizes exceeding 8 µm are sufficient to facilitate the complete diffusion of nutrients and metabolites necessary for the proliferation of hADSCs [41], a qualification that our hydrogel also satisfies. The microstructure of freeze-dried chitosan–PEG/PEG–dialdehyde hydrogels were characterized using a scanning electron microscope (SEM). The hydrogels showed a continuous and porous structure with pore diameters ranging from 20 to 50 µm (Figure 2d). Also, the hydrogels exhibited notable self-healing properties (Figure 2e). The self-healing mechanism of chitosan–PEG/PEG–dialdehyde hydrogels relies on the presence of dynamic covalent bonds, particularly imide bonds formed via the Schiff base reaction [42]. These inherent self-healing properties of an injectable polymer hydrogel offer a critical advancement in biomedical applications, allowing hydrogels to autonomously repair damage and restore their structural integrity to enhance therapeutic efficacy.

#### 2.2.2. Rheological Analysis of Hydrogels

To optimize the mechanical properties of hydrogels for the support of stem cell proliferation and differentiation, rheological analysis was conducted on both pre-crosslinked and post-crosslinked samples. The hydrogel was formulated by gently blending chitosan–PEG (2.5%, *w*/*v*) and PEG–dialdehyde (1.0 g/mL) at a volume ratio of 6:1 using a previously mentioned double-barrel syringe. Subsequently, the temporal changes in storage modulus (G’) and loss modulus (G”) during gelation were examined to elucidate the mechanical attributes of the crosslinked hydrogel.

**Figure 3:**
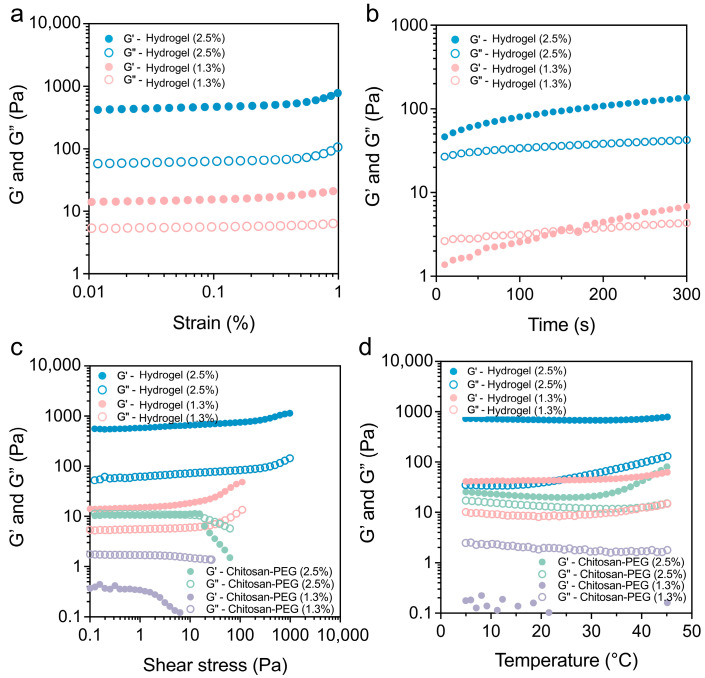
Rheological analysis of chitosan–PEG/PEG–dialdehyde hydrogels. (**a**) The storage modulus (G’, filled symbols) and loss modulus (G”, empty symbols) were recorded under the strain sweep at 37 °C and 1 Hz for chitosan–PEG/PEG–dialdehyde hydrogels with different concentrations (1.3% and 2.5%, *w*/*v*), showing that strain at 0.1% was within the linear viscoelastic regime (LVR) with less noise. (**b**) The G’ and G” were recorded under the time sweep for chitosan–PEG/PEG–dialdehyde hydrogels with different concentrations (1.3% and 2.5%, *w*/*v*) right after injection, showing the crossover point of G’ and G” for verification of gelation (G’ > G”). (**c**) The G’ and G” were recorded under increasing shear stress for chitosan–PEG and chitosan–PEG/PEG–dialdehyde hydrogels with different concentrations (1.3% and 2.5%, *w*/*v*) at 37 °C to compare the strength of different hydrogels. (**d**) The G’ and G” were recorded under increasing temperatures for chitosan–PEG and chitosan–PEG/PEG–dialdehyde hydrogels with different concentrations (1.3% and 2.5%, *w*/*v*) at 37 °C to show their thermosensitivity.

To determine the appropriate strain and frequency settings for time-sweep experiments in rheological analysis to test gelation time, chitosan–PEG/PEG–dialdehyde hydrogels at different concentrations (1.3% and 2.5%, *w*/*v*) were subjected to incremental strain at 1 Hz and 37 °C. The results in Figure 3a revealed that within the range of 0.01–0.3% strain, the material exhibited behavior within the linear viscoelastic regime (LVR), where the G’ remained independent of the applied strain. Consequently, a strain of 0.1% at 1 Hz was selected as the testing condition to minimize hydrogel damage and mitigate noise during the time sweep analysis.

Rapid gelation is crucial for preserving cell viability, maintaining spatial integrity, and fostering effective cellular interactions, ultimately enhancing the success of the tissue regeneration process. To investigate the gelation time as well as the change of modulus over time post-injection, the G’ and G” of freshly mixed chitosan–PEG/PEG–dialdehyde hydrogels were monitored at 37 °C (Figure 3b). In the case of the chitosan–PEG/PEG–dialdehyde hydrogel at a higher concentration (2.5%, *w*/*v*), the hydrogel exhibited a dominance of G’ over loss modulus G”. There was no crossover point between G’ and G”, suggesting an instantaneous gelation upon injection. Both G’ and G” exhibited an increasing trend over time, accompanied by a widening discrepancy between them, indicating the ongoing progression of crosslinking processes (Figure 3b). For the chitosan–PEG/PEG–dialdehyde hydrogel at a lower concentration (1.3%, *w*/*v*), the crossover point between G’ and G” manifested around 150 s (Figure 3b). These results reveal a direct correlation between the concentration of the chitosan–PEG polymer and gelation time. Furthermore, the modulus of the hydrogel can be precisely tailored by adjusting the concentration of the chitosan–PEG polymer, providing versatility for a range of applications.

The structural integrity of the chitosan–PEG/PEG–dialdehyde hydrogel was assessed through stress sweep testing, comparing it to the chitosan–PEG without crosslinker at different concentrations at 1 Hz and 37 °C. The results in Figure 3c show that G’ was smaller than G” for chitosan–PEG at 1.3%, *w*/*v* without crosslinker, indicating that the chitosan–PEG did not form a hydrogel at 37 °C at this concentration. On the contrary, a comparable value of G’ and G” was found for chitosan–PEG at 2.5%, *w*/*v* without crosslinker, indicating that higher polymer concentrations can increase the elasticity of chitosan–PEG. Also, at 2.5% (*w*/*v*), both the G’ and G” of the chitosan–PEG sample remained stable as stress increased, showing no significant changes until reaching approximately 10 Pa. Beyond this threshold, the G’ and G” sharply declined, indicating that the sample experienced significant structural damage. After adding the PEG–dialdehyde, the chitosan–PEG transform into chitosan–PEG/PEG–dialdehyde hydrogels (G’ > G”) at both concentrations (Figure 3c). The results revealed that the concentration of chitosan–PEG affected the strength of the formed chitosan–PEG/PEG–dialdehyde hydrogel when the amount of PEG–dialdehyde crosslinker was held constant.

Moreover, the storage modulus of chitosan–PEG/PEG–dialdehyde hydrogels at 2.5% *w*/*v* consistently surpassed that of hydrogels at 1.3% *w*/*v*. At 2.5% (*w*/*v*), the limit of the LVR of hydrogels was increased to approximately 900 Pa, with G’ reaching approximately 1000 Pa, indicating the hydrogels maintain structural integrity against higher stresses compared to the uncrosslinked samples. Importantly, previous studies indicated that hydrogels with a storage modulus in the range of 500–1000 Pa were optimal for cartilage regeneration, whereas hydrogels with a storage modulus around 3000 Pa were more suitable for fibrous tissue repair [43]. Therefore, chitosan–PEG/PEG–dialdehyde hydrogels (2.5%, *w*/*v*) were chosen as the optimized gel for subsequent cell experiments.

To further substantiate that the Schiff base reaction can specifically induce gelation between chitosan–PEG and modified PEG 2000 (PEG–dialdehyde), a sample created by blending chitosan–PEG with unmodified PEG 2000 (PEG–OH), denoted as chitosan–PEG/PEG–OH, was included as a reference. In the case of chitosan–PEG/PEG–OH, despite the absence of chemical crosslinking interactions between chitosan–PEG and PEG–OH, the mixture displayed a G’ larger than the G” independent of the applied stress reaching approximately 70 Pa, which is much lower than that of chitosan–PEG/PEG–dialdehyde (900 Pa). This observation suggests the formation of a gel-like chitosan–PEG/PEG–OH mixture (Figure S2). The occurrence can be ascribed to the highly hydrophilic nature of PEG–OH, facilitating the extraction of water molecules from chitosan–PEG. Consequently, hydrophobic interactions originating from chitosan take precedence, triggering a sol–gel transition. However, the chitosan–PEG/PEG–OH hydrogel exhibited notably weak mechanical strength (G’ < 20 Pa), rendering it unsuitable for cell-laden hydrogel-based therapy [8,35].

Thermosensitive hydrogels can undergo reversible phase transitions in response to temperature changes, offering precise control over drug delivery, tissue engineering, and various biomedical applications. Earlier studies have demonstrated that the chitosan–PEG solution displays thermosensitive gelation without adding covalent crosslinkers. The temperature at which gelation occurs can be controlled by manipulating the grafting ratio of the hydrophilic PEG side chain [44,45,46]. The disruption of hydrogen bonds between water and PEG, resulting in the dominance of hydrophobic interactions originating from chitosan, accelerates the sol–gel transition with increasing temperature. This transformation converts the solution into a hydrogel state [40,44].

Here, chitosan–PEG at a lower concentration (1.3%, *w*/*v*) did not undergo hydrogel formation within the temperature range of 5 °C to 45 °C, and its modulus exhibited no temperature dependence. Conversely, at a higher concentration (2.5%, *w*/*v*), chitosan–PEG exhibited hydrogel-like behavior, characterized by a significant increase in G’ beyond 30 °C. For chitosan–PEG/PEG–dialdehyde hydrogels at both concentrations (1.3% and 2.5%, *w*/*v*), G’ remained consistent below 30 °C, with a slight increase beyond 30 °C compared to chitosan–PEG (Figure 3d), due to the hydrophobic induced crosslinking. This behavior can be attributed to chemical crosslinking, which restricts hydrophobic interactions between molecular chains. Therefore, chitosan–PEG/PEG–dialdehyde demonstrated double crosslinking network behavior at 37 °C, involving both Schiff base chemical crosslinking and hydrophobic physical crosslinking.

#### 2.2.3. Swelling and Degradation of Hydrogels

**Figure 4:**
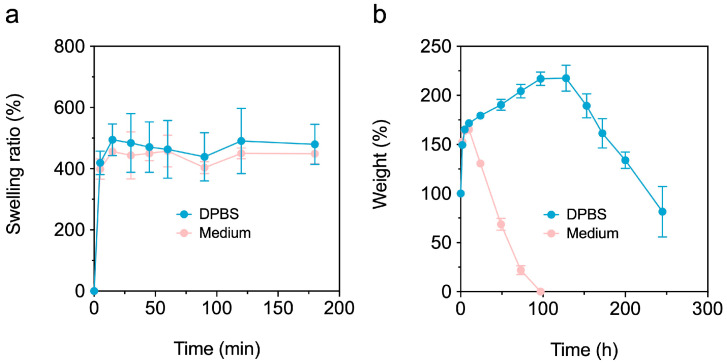
Hydrogel swelling and degradation behaviors. (**a**) Swelling ratio and (**b**) degradation of chitosan–PEG/PEG–dialdehyde hydrogels incubated in complete human mesenchymal stem cell medium or DPBS and maintained at 37 °C. The hydrogels were prepared by mixing chitosan–PEG (2.5%, *w*/*v*) and PEG–dialdehyde (1 g/mL) at a volume mixing ratio of 6:1.

The swelling behavior of a cell-laden hydrogel is important as it directly influences the transport of nutrients, oxygen, and signaling molecules essential for cell viability and tissue regeneration. A well-controlled and optimized swelling response ensures a microenvironment that promotes cellular interactions, proliferation, and ultimately enhances the success of tissue regeneration in hydrogel-based therapies. Figure 4a illustrates the equilibrium swelling ratio of freeze-dried chitosan–PEG/PEG–dialdehyde (2.5%, *w*/*v*) composite hydrogels, as determined in complete human mesenchymal stem cell (hADSC) medium or Dulbecco’s phosphate-buffered saline (DPBS). The freeze-dried hydrogel exhibited rapid water absorption, reaching equilibrium within the initial 15 min. Notably, the swelling ratio in the hADSC medium closely mirrored that in DPBS, both approximating ~500%, and no substantial difference was discerned between them over 3 h. This rapid swelling behavior signifies the hydrogel’s ability to swiftly attain equilibrium post-injection, facilitating stable water retention and the establishment of an optimal microenvironment. This ensures sustained nutrient supply, oxygen diffusion, and efficient removal of metabolic byproducts, thereby creating favorable conditions for mesenchymal stem cell proliferation and tissue regeneration.

The degradation behavior of a cell-laden hydrogel is pivotal as it governs the temporal release of encapsulated bioactive molecules, facilitates cell migration, and allows for the integration of newly formed tissue with the surrounding environment. A controlled and tailored degradation profile ensures harmonious coordination between hydrogel breakdown and tissue regeneration processes, maximizing the therapeutic efficacy and long-term success of hydrogel-based strategies in tissue engineering and regenerative medicine. The degradation kinetics of chitosan–PEG/PEG–dialdehyde (2.5%, *w*/*v*) composite hydrogels were systematically evaluated over time by incubating them in both hADSC medium and DPBS at 37 °C (Figure 4b). The weight of freshly prepared hydrogels in hADSC medium initially increased in the first 5 h due to swelling. However, by day 5, these hydrogels had undergone complete degradation. In contrast, the weight of hydrogels in DPBS exhibited a slower increase in the initial 5 days, followed by a subsequent decrease. These hydrogels retained over 80% of their initial weight even after 10 days, indicating a comparatively slower degradation rate. This nuanced understanding of degradation behavior is pivotal for tailoring hydrogel formulations to achieve optimal temporal release profiles in cell-laden environments, enhancing their potential for controlled and sustained therapeutic applications.

### 2.3. Morphology and Proliferation of hADSCs

Evaluating the morphology and proliferation of encapsulated cells provides insights into the three-dimensional growth dynamics and viability of cells in hydrogels. These assessments are instrumental for understanding the effectiveness of the hydrogel platform in supporting cellular behavior, offering essential information for the development and optimization of stem cell-based therapies for enhanced tissue regeneration. Although stem cell therapy has exhibited promising results in osteoarthritis treatment, its long-term application is constrained by challenges such as limited cell survival, extensive cell death, poor cellular function, and inadequate distribution post-injection. Softer hydrogels with a stiffness below 1000 Pa have been recognized for their capacity to preserve stem cell viability, proliferation, and stemness [47,48,49], mitigating issues associated with transplanted cell death and enhancing the therapeutic efficacy of targeted stem cell injections [50]. As shown in the earlier discussion, chitosan–PEG/PEG–dialdehyde (2.5%, *w*/*v*) hydrogels have a storage modulus of less than 1000 Pa, indicating that this soft hydrogel is promising for the treatment of osteoarthritis.

**Figure 5:**
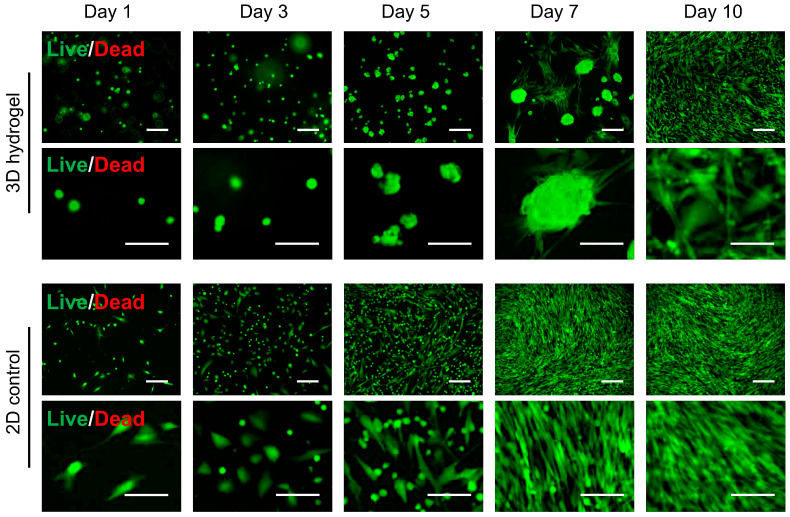
The effect of culture environment on hADSC viability. Fluorescence images of hADSCs cultured in chitosan–PEG/PEG–dialdehyde hydrogels (2.5%, *w*/*v*, 3D hydrogel) and on regular 2D surfaces (2D control) on days 1, 3, 5, 7, and 10. hADSCs were stained with Calcein-AM (green, for live) and propidium iodide (red, for dead). Short scale bar represents 200 μm, and long scale bar represents 100 μm.

We then investigated the potential of the hydrogels to sustain the viability and proliferation of stem cells. The morphology of hADSCs was examined using a microscope and the viability was tracked using fluorescence imaging. The proliferation was assessed through an alamarBlue assay. Illustrated in Figure 5 and Figure S3 is the growth trajectory of hADSCs in chitosan–PEG/PEG–dialdehyde (2.5%, *w*/*v*) hydrogels (3D hydrogel) in comparison to their 2D counterparts on conventional tissue culture plates (TCPS) over 10 days. From day 1 to day 5, the gel-encapsulated cells started proliferating, leading to the gradual formation of increasingly larger cell spheroids within the hydrogels, and the density of spheroids increased. By day 7, stem cell spheroids began adhering and proliferating on the two-dimensional surface, ultimately achieving complete adherence on day 10 due to hydrogel degradation. Notably, over these 10 days, cell viability consistently surpassed 98%.

Furthermore, the injection process conducted using the double-barrel syringe did not compromise the viability of stem cells, as depicted in Figure S4. These results collectively highlight the capability of chitosan–PEG/PEG–dialdehyde hydrogels (2.5%, *w*/*v*) as a stem cell delivery platform to sustain high viability and proliferation.

**Figure 6:**
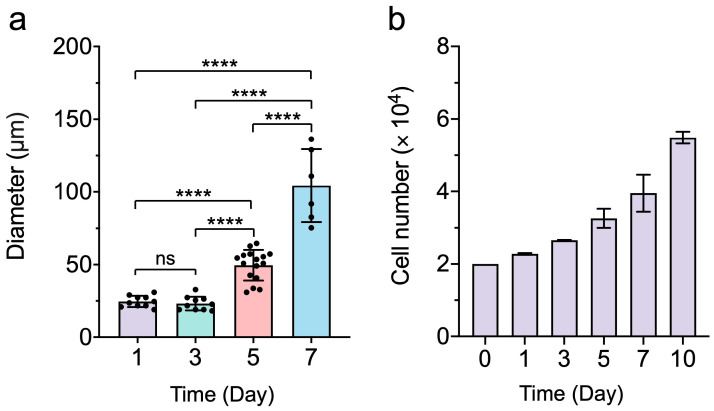
(**a**) The diameter of cell spheroids in chitosan–PEG/PEG–dialdehyde hydrogels (2.5%, *w*/*v*) on day 1, 3, 5, and 7. One-way ANOVA followed by Tukey’s multiple comparison test was used to determine whether the differences were statistically significant (mean ± SD, *n* > 5; **** *p* < 0.0001; ns, nonsignificant). (**b**) Cell proliferation results tested by the alamarBlue assay on day 1, 3, 5, 7, and 10 days for hADSCs cultured in chitosan–PEG/PEG–dialdehyde hydrogels (2.5%, *w*/*v*) (*n* = 3).

Also, during the initial 3-day culture period in chitosan–PEG/PEG–dialdehyde (2.5%, *w*/*v*) hydrogels, the size of cell spheroids maintained a consistent dimension of approximately 25 µm (Figure 6a), indicating a phase of relative stability. Subsequently, a substantial increase in spheroid size was observed from day 3 to day 7, reaching a size of approximately 100 µm. Concurrently, the cell number exhibited a remarkable growth trajectory throughout the 10-day culture period, escalating from an initial count of 20,000 to 57,000 (Figure 6b). These observations signify a dynamic cellular response within the hydrogels, where the initial phase is characterized by a maintained spheroid size, followed by a significant expansion. Importantly, the steady increase in cell number over time indicates the hydrogels’ capacity to support cell proliferation during the degradation process. Furthermore, it demonstrates that cells encapsulated in the hydrogels can thrive and proliferate robustly after the gel has degraded and cells have been released, confirming the regenerative potential of the chitosan–PEG/PEG–dialdehyde hydrogel (2.5%, *w*/*v*) platform.

### 2.4. Expression of Genes Relevant to Cartilage Regeneration

To investigate the potential of chitosan–PEG/PEG–dialdehyde hydrogels for cartilage tissue regeneration, we induced chondrogenesis in the encapsulated stem cells and assessed the expression of chondrogenesis-related genes, including ACAN (Aggrecan), SOX9 (SRY-box transcription factor 9), and Col-II (Collagen Type II). ACAN is essential for cartilage integrity, encoding the aggrecan protein that forms the core of the cartilage extracellular matrix, providing hydration and resistance. SOX9, a transcription factor, orchestrates chondrocyte differentiation and cartilage formation by regulating the expression of genes such as ACAN and Col-II. Col-II, a key constituent of the cartilage matrix, imparts structural stability and strength.

**Figure 7:**
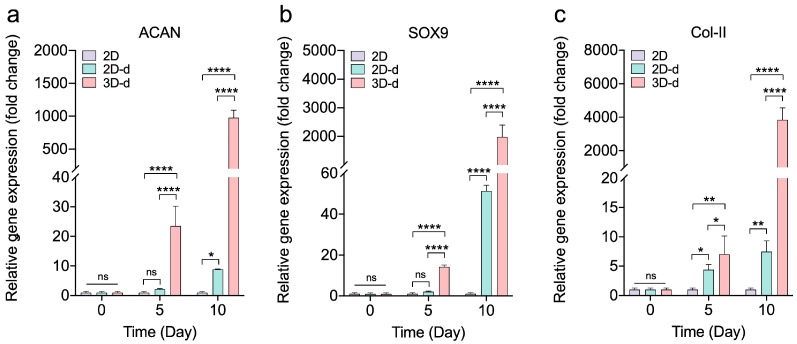
Gene expression associated with chondrogenic differentiation. Relative expression of RNA: (**a**) ACAN, (**b**) SOX9, and (**c**) Col-II contents in hADSCs cultured on 2D TCPS and in 3D hydrogels in chondrogenic differentiation medium for a 10-day culture period. Gene expression was first normalized to glyceraldehyde 3-phosphate dehydrogenase (GAPDH) (reference gene) and then normalized to the expression associated with 2D TCPS culture on day 0 (set as 1-time). One-way ANOVA followed by Tukey’s multiple comparison test was used to determine whether the differences were statistically significant (mean ± SD, *n* = 3; * *p* < 0.05, ** *p* < 0.01, **** *p* < 0.0001; ns, nonsignificant). Legend: 2D = hADSCs cultured on 2D TCPS in the complete cell culture medium, 2D-d = hADSCs cultured on 2D TCPS in the chondrogenic differentiation medium, 3D-d = hADSCs cultured in 3D hydrogels in the chondrogenic differentiation medium.

In this study, we examined the relative expression of these crucial genes under diverse culture conditions. Specifically, cells were cultured on conventional 2D TCPS and in 3D hydrogels using a chondrogenic differentiation medium. Figure 7 illustrates that the 3D hydrogel platform enhances the expression of genes associated with chondrogenic differentiation compared to the 2D TCPS surface on both day 5 and day 10. This suggests that the 3D hydrogel better mimics the native cartilage microenvironment, offering superior mechanical support and structural cues, facilitating cellular organization and interaction, and ultimately boosting the chondrocyte maturation for cartilage regeneration. These findings demonstrated the potential of the hydrogel system as a promising platform for effective cartilage regeneration, emphasizing the vital role of microenvironmental factors in directing chondrogenic differentiation and tissue development.

### 2.5. Expression of Proteins Relevant to Cartilage Regeneration

**Figure 8:**
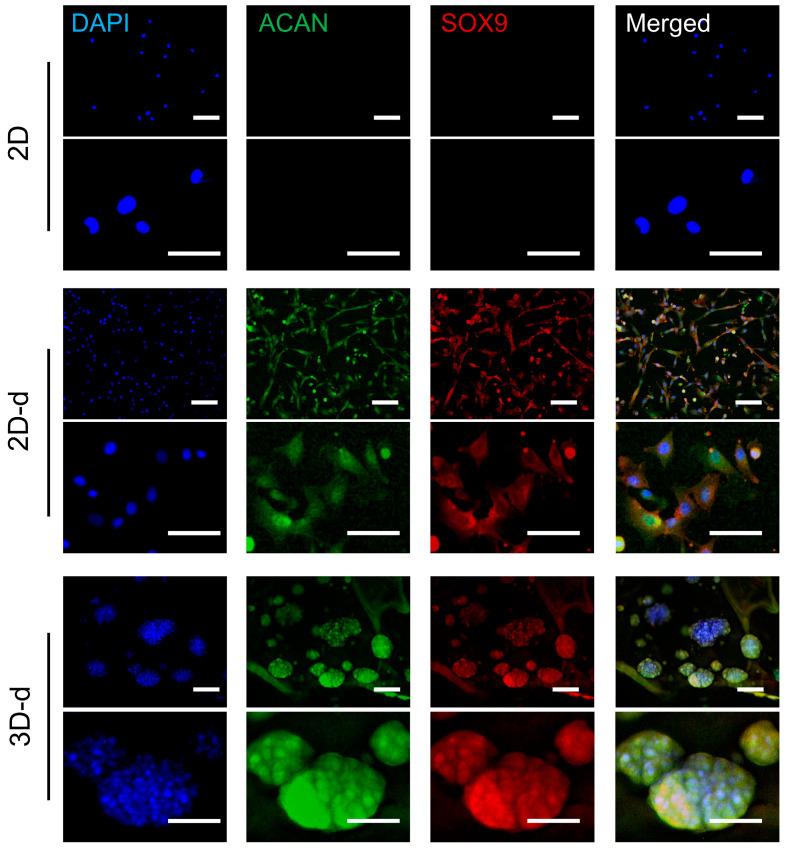
Protein expression associated with chondrogenic differentiation of hADSCs encapsulated in hydrogels. Immunofluorescence staining was applied for Aggrecan (ACAN, green), SRY-Box Transcription Factor 9 (SOX9, red), and cell nuclei (DAPI, blue). hADSCs were cultured in chitosan–PEG/PEG–dialdehyde hydrogels (2.5%, *w*/*v*) in the chondrogenic differentiation medium (3D-d) for a 10-day culture period. hADSCs cultured on 2D TCPS surface in both chondrogenic differentiation medium (2D-d) and normal cell culture medium (2D) were used as a control. The short scale bar represents 200 μm, and the long scale bar represents 100 μm.

We used immunofluorescence staining to evaluate the expression of specific proteins that serve as markers for chondrogenic differentiation of the hADSCs encapsulated in chitosan–PEG/PEG–dialdehyde hydrogels. The expression of chondrogenic markers, ACAN and SOX9 proteins, were visualized by green and red fluorescence, respectively. Cell nuclei were concurrently counterstained with DAPI in blue. Figure 8 illustrates that hADSCs cultured in 3D hydrogels (3D-d) and on a 2D surface in a chondrogenic differentiation medium (2D-d) consistently exhibited robust expression of ACAN (green) and SOX9 (red). Conversely, hADSCs cultured on the 2D surface in a complete cell culture medium (2D) exhibited restricted expression of ACAN and SOX9, to the extent that fluorescence was not observable. Although hADSCs cultured in both 3D-d and 2D-d environments exhibited the expression of chondrogenic differentiation-associated proteins, specifically ACAN and SOX9, the distinct advantages of 3D hydrogels over 2D surfaces should be emphasized. The 3D hydrogels better mimic the native tissue microenvironment, providing essential spatial cues and mechanical support crucial for cellular organization and function. Unlike 2D surfaces, which limit cell–cell and cell–matrix interactions, 3D hydrogels facilitate multidirectional interactions between cells and the surrounding matrix, thereby promoting physiological responses and offering a more accurate representation of in vivo conditions. Furthermore, the porous structure of hydrogels enhances the diffusion of nutrients and signaling molecules, which improves cellular behavior and promotes the development of complex tissues. These strongly suggest that the 3D hydrogel environment effectively supports the chondrogenic differentiation of hADSCs.

## 3. Conclusions

This study presents an innovative approach for the in situ creation of a biological compatible, biodegradable, injectable, and self-healing polysaccharide hydrogel. This hydrogel holds advantages in minimally invasive delivery and sustained support of human adipose-derived stem cells with high viability, demonstrating its potential for effective cartilage regeneration. The gelation is achieved by mixing chitosan–PEG and PEG–dialdehyde, resulting in a homogeneous and highly porous hydrogel network crosslinked by both Schiff base reactions and hydrophobic interactions. By precisely controlling the concentrations of the polymer solutions, we were able to customize the hydrogel to achieve the desired mechanical properties, gelation time, and degradation rate, ensuring the sustained viability of the encapsulated cells. Both the encapsulated and released cells remained highly viable for at least 10 days post-injection, affirming the hydrogel’s suitability for supporting cartilage regeneration. Furthermore, the cells encapsulated in the hydrogels formed cell spheroids and exhibited robust chondrogenesis reflected on both gene expression and protein levels. This injectable hydrogel, laden with stem cells, holds promise for cartilage regeneration as well as applications in other cell-based therapies.

## 4. Materials and Methods

### 4.1. Materials

Chitosan (medium molecular weight, 75–85% deacetylated), poly(ethylene glycol) 2000 (PEG 2000), poly(ethylene glycol) methyl ether (Mn 750) (mPEG), succinic anhydride, acetic anhydride, N-(3-Dimethylaminopropyl)-N′-ethylcarbodiimide hydrochloride (EDC), and N-hydroxysuccinimide (NHS) were purchased from Sigma-Aldrich (St. Louis, MO, USA). Calcein AM, propidium iodide, alamarBlue cell viability reagent, aggrecan monoclonal antibody (BC-3), SOX9 recombinant rabbit monoclonal antibody (7H13L8), goat anti-mouse IgG, IgM (H + L) secondary antibody with Alexa Fluor™ 488, and donkey anti-rabbit IgG (H + L) highly cross-adsorbed secondary antibody with Alexa Fluor™ 555 were purchased from Thermo Fisher Scientific Inc. (Waltham, MA, USA). Human adipose-derived mesenchymal stem cells (hADSCs), MSC cell culture medium, fetal bovine serum (FBS), penicillin/streptomycin solution (P/S solution), and mesenchymal stem cell growth supplement were purchased from ScienCell Research Laboratories (Carlsbad, CA, USA). All chemicals and reagents were used as received.

### 4.2. Synthesis of Chitosan–PEG

The chitosan–PEG was synthesized through amide linkage formation between chitosan and mPEG as previously reported [40]. Briefly, first, the chitosan powder was purified by successively dissolving into 0.1 M acetic acid and 1 M NH_4_OH to remove the impurities through centrifugation. The purified chitosan was washed with DI water, lyophilized, and stored at –20 °C before modification. Second, the terminal hydroxy groups of mPEG were substituted with carboxylic acid groups through the ring-open reaction of succinic anhydride to mPEG to form mPEG–acid. Third, mPEG–acid (0.43 g) was reacted with purified chitosan (0.35 g) in DI containing 0.33% *v*/*v* acetic acid using EDC (0.2 g)/NHS (0.12 g) coupling chemistry at 45 °C for 4 h. After that, 0.5 M NaOH solution was added to reach pH 7.0. The solution was dialyzed (MW 3.5K cutoff) five times against deionized (DI) water and lyophilized. An ^1^H NMR (AV-500, Bruker, Karlsruhe, Germany) was used to analyze the chemical structures of purified polymers, which were preserved at –20 °C.

### 4.3. Synthesis of PEG–Dialdehyde

The synthesis of PEG–dialdehyde involved the oxidation of PEG 2000 using a combination of dimethyl sulfoxide (DMSO) and acetic anhydride. Initially, a mixture of 10 mL acetic anhydride and 50 mL DMSO/chloroform (9/1, *v*/*v*) was prepared. Subsequently, 5 g of PEG 2000 powder was added to the solution and completely dissolved at 25 °C. The reaction took place under a nitrogen atmosphere for 15 h at room temperature. Next, the PEG–dialdehyde was precipitated using diethyl ether, redissolved in chloroform, and subjected to three cycles of dissolution and precipitation. The resulting precipitated samples were dissolved in DI water and then freeze-dried. The final product obtained was a white powder of PEG–dialdehyde, which was stored at –20 °C. The chemical structure of the purified PEG–dialdehyde was analyzed using ^1^H NMR spectroscopy (AV-500, Bruker, Karlsruhe, Germany).

### 4.4. Fabrication of Hydrogels

The chitosan–PEG was dissolved into DPBS or the complete human mesenchymal stem cell medium (5% of FBS, 1% of mesenchymal stem cell growth supplement, and 1% of penicillin/streptomycin in basal medium) on ice, which was labeled as solution (a). The PEG–dialdehyde was dissolved into DPBS at 1 g/mL at room temperature, which was labeled as solution (b). Solution (a) and (b) were gently blended (6:1, *v*/*v*) using a medical-grade double-barrel syringe (Duploject syringe, Baxter Healthcare, Deerfield, IL, USA) with a 21 G needle (Becton & Dickinson, Franklin Lakes, NJ, USA) to produce the chitosan–PEG/PEG–dialdehyde hydrogel spontaneously. All polymer solutions were sterilized by filtration through 0.2 μm membrane filters prior to hydrogel preparation.

### 4.5. Hydrogel Morphologies

For morphological analysis, the solvent was substituted with distilled water to generate hydrogels. The prepared hydrogels were quickly frozen by liquid nitrogen and further lyophilized. The resulting lyophilized hydrogels were examined for both surface and cross-sectional morphologies using a SEM operating at an acceleration voltage of 15 kV (Hitachi TM3000 Tabletop Microscope, Hitachi High-Technologies Corporation, Tokyo, Japan). The pore size was determined by analyzing SEM images using ImageJ (version 1.53).

### 4.6. Hydrogel Viscoelasticity

The viscoelastic properties and the modulus of uncrosslinked and crosslinked chitosan hydrogels were characterized using a stress-controlled rheometer (Anton Paar MCR 92, Anton Paar GmbH, Graz, Austria). The rheological properties of crosslinked chitosan–PEG/PEG–dialdehyde (chitosan–PEG/PEG–dialdehyde) at concentrations of 1.3% and 2.5% (*w*/*v*) were investigated. The strain sweep was conducted in a dynamic oscillatory mode with a constant 1 Hz frequency. Storage modulus (G’) and loss modulus (G”) were recorded as a function of strain from 0.01% to 1% at 37 °C. Strain at 0.1% and 1 Hz was chosen for subsequent time sweep measurements in a dynamic oscillatory mode where chitosan–PEG and PEG–dialdehyde solutions were injected into the measurement stage and the measurements began immediately. Storage modulus (G’) and loss modulus (G”) were recorded as a function of time every 10 s for more than 5 min at 37 °C to investigate the evolution during crosslinking formation. The strength of the uncrosslinked chitosan–PEG solution and crosslinked chitosan–PEG/PEG–dialdehyde hydrogels at concentrations of 1.3% and 2.5% (*w*/*v*) were evaluated via stress sweep at 37 °C and 1 Hz. The storage modulus (G’) and loss modulus (G”) were recorded as a function of stress from 0.1 to 10,000 Pa. The thermogelling properties of the uncrosslinked chitosan–PEG solution and crosslinked chitosan–PEG/PEG–dialdehyde hydrogels at concentrations of 1.3% and 2.5% (*w*/*v*) were evaluated by measuring storage modulus (G’) and loss modulus (G”) with increasing temperature. Frequency (1 Hz) and strain (0.1%) were applied, and the temperature was increased from 5 to 45 °C at 1 °C min^–1^.

### 4.7. Hydrogel Equilibrium Swelling

Freeze-dried hydrogels of known weights were immersed in complete human mesenchymal stem cell medium or DPBS and maintained at 37 °C. At different time points, the swollen hydrogels were collected and promptly weighed with a microbalance. The following equation was used to calculate the equilibrium swelling ratio (ESR): ESR = (W_t_ − W_0_)/W_0_ × 100%, where W_t_ and W_0_ are the weights of the hydrogels at the swelling state and at the dry state, respectively.

### 4.8. Hydrogel Degradation In Vitro

The weight loss caused by the degradation of hydrogels was also investigated. After a gentle blending process, the produced chitosan–PEG/PEG–dialdehyde hydrogels were cured at 4 °C for 24 h to ensure full gelation. The hydrogels of known weights were immersed in complete human mesenchymal stem cell medium or DPBS and maintained at 37 °C. At different time points, the hydrogels were collected and promptly weighed with a microbalance. The value of W_t_/W_0_ × 100% was used to calculate the weight remaining ratio, where W_t_ and W_0_ are the weights of the hydrogels at different time points and at the initial state, respectively.

### 4.9. Cell Culture

Human adipose-derived mesenchymal stem cells (hADSCs), fetal bovine serum (FBS), mesenchymal stem cell growth supplement, and penicillin/streptomycin solution were purchased from ScienCell Research Laboratories (Carlsbad, CA, USA). hADSCs were seeded in a polystyrene tissue culture dish (diameter = 6 cm) at a density of 5.0 × 10^4^ cells/mL in complete human mesenchymal stem cell medium (5% of FBS, 1% of mesenchymal stem cell growth supplement, and 1% of penicillin/streptomycin in basal medium) at 37 °C in a humidified atmosphere containing 5.0% CO_2_. Subconfluent cell cultures were passaged using 0.25% trypsin/EDTA.

### 4.10. Live/Dead Staining

Calcein-AM (Thermofisher, Waltham, MA, USA) and propidium iodide (Thermofisher, Waltham, MA, USA) were used to stain live and dead cells, respectively. Calcein-AM was dissolved in DMSO to obtain a stock concentration of 1 mM, and propidium iodide was dissolved in DI water to obtain a stock concentration of 1.5 mM. The chitosan–PEG was dissolved into the complete human mesenchymal stem cell medium on ice at 3.0% (*w*/*v*). The PEG–dialdehyde was dissolved into DPBS at 1.0 g/mL at room temperature. The desired amount of hADSCs were suspended into a complete human mesenchymal stem cell medium and the suspension solution was gently mixed with the chitosan–PEG solution (1/5, *v*/*v*). Subsequently, this solution was gently mixed with PEG–dialdehyde solution (6:1, *v*/*v*) to produce hydrogels. Finally, a three-dimensional hydrogel encapsulating hADSCs was spontaneously formed. To evaluate the viability of encapsulated hADSCs, 112 µL of hydrogels containing 2.0 × 10^4^ cells was loaded into each well of 96-well plate. Then, 150 µL of complete human mesenchymal stem cell medium was added onto the top of gels and the medium was changed every day. At different time points (day 1, 3, 5, 7, and 10), 100 µL of DPBS solution containing calcein AM and propidium iodide (1.5 mM) was added into each well and light-proof incubated for 15 min at room temperature. The morphologies of cells were obtained using a fluorescence phase-contrast microscope (model IX 81; Olympus, Tokyo, Japan). As a control, 1000 cells in 200 µL of complete human mesenchymal stem cell medium were seeded into each well of 96-well plate. In addition, the effect of the injection process on hADSC viability was also evaluated using the Live/Dead staining method.

### 4.11. Cell Proliferation Test

The alamarBlue assay was used to assess the proliferation and viability of hADSCs encapsulated in hydrogels using the same method mentioned in the Live/Dead staining section. At each time point, 150 µL of complete cell culture medium containing 10% alamarBlue solution was added after removing the cell culture medium and incubated at 37 °C for 3 h, followed by transferring 100 µL of the alamarBlue solution to a black 96-well plate. Measurements were performed using a plate reader (Spectra Max M2, Molecular Devices, Union City, CA, USA), and the fluorescence intensities at 590 nm were obtained at an excitation wavelength of 560 nm.

### 4.12. cDNA Synthesis and Quantitative Reverse Transcriptase Polymerase Chain Reaction (qRT-PCR)

To analyze the relative gene expression, qRT-PCR was performed on cDNA collected from cells cultured in both hydrogels and 2D surfaces after 5 days and 10 days of growth. The Qiagen RNeasy kit (Qiagen, Valencia, CA, USA) was used to extract ribonucleic acid (RNA) from cells and the iScript cDNA synthesis kit was used to make cDNA according to the manufacturer’s instructions. In a Bio-Rad CFX96 real-time PCR detection system (Bio-Rad Laboratories, Inc. Hercules, CA, USA), the amplification with a primer for each of the transcripts was evaluated using SYBR Green PCR Mastermix. The reference gene was glyceraldehyde 3-phosphate dehydrogenase (GAPDH). The cycle number at threshold fluorescence intensity was used to determine quantification. All samples were thermocycled in a 10 µL solution comprising 5 µL SYBR Mastermix, 300 nm primers (Table S1, Integrated DNA Technologies, Coralville, IA), and cDNA at 0.04–1 ng/µL concentrations that were adjusted for each condition. The thermocycle proceeded at 95 °C for 2 min, followed by 40 cycles of denaturation at 95 °C for 15 s, annealing at 58 °C for 30 **s**, and extension at 72 °C for 30 s. The CFX Manager program was used to examine all RT-qPCR results (Bio-Rad Laboratories, Inc., Hercules, CA, USA).

### 4.13. Immunofluorescence and Immunocytochemistry

The samples were prepared using the identical procedure as for the Live/Dead staining method. Specifically, 96 µL of the chitosan–PEG solution (2.5%, *w*/*v*) was mixed with 16 μL of PEG–dialdehyde solution (1 g/mL) in each well of an 8-well chamber slide. The total volume of hydrogel in each well was 112 μL, and the total number of hADSCs in each well was 20,000. The experiment for each concentration was repeated three times. Subsequently, the cells were cultivated in a medium designed for chondrogenic differentiation for 14 days, and the medium was changed every two days. The cells in the hydrogel were fixed with a 4% paraformaldehyde solution for 15 min at room temperature. Following fixation, the cells were permeabilized with 1% (*v*/*v*) Triton X-100 in DPBS and blocked with 15% donkey serum albumin and 0.3% Triton X-100 in DPBS for 1 h at room temperature. The cells were then incubated with primary antibodies ACAN and SOX9 overnight at 4 °C, followed by incubation with the corresponding secondary antibodies for 1 h at room temperature in the dark. The nuclei were counterstained with 1 μM DAPI for 15 min. Between these steps, thorough washing with cold PBS was carried out at least three times to remove any unbound antibodies or other reagents. All the immunostained samples were imaged using a Nikon TE300 inverted microscope (Nikon Corporation, Tokyo, Japan).

### 4.14. Statistical Analysis

All graphs and bar charts are expressed as the mean ± standard deviation (SD) of more than three repeated experiments as described above. The mean values of the diameter of cell spheroids and the relative expression of RNA were compared by one-factor analysis of variance (ANOVA) and the significance of the difference was determined by Tukey’s multiple comparisons test.

## Data Availability

The data that support the findings of this study are available from the corresponding author upon reasonable request.

## Supplementary Materials

The following supporting information can be downloaded at: https://www.mdpi.com/article/10.3390/gels10080508/s1, Figure S1: Shear thinning property and injectability of chitosan–PEG/PEG–dialdehyde hydrogel; Figure S2: Rheological analysis of chitosan–PEG and chitosan–PEG/PEG–OH mixture; Figure S3: The morphology of hADSCs over time; Figure S4: The effect of injection process on hADSCs viability; Table S1: Primer sequences of the reference and target genes.
