# Supplementary material for: Injectable Biodegradable Chitosan–PEG/PEG–Dialdehyde Hydrogel for Stem Cell Delivery and Cartilage Regeneration
Source: Gels. 2024 Aug 1;10(8):508. doi: 10.3390/gels10080508 (PMC11353310; doi:10.3390/gels10080508)
Supplement: Supplementary file 1 [file gels-10-00508-s001.zip › gels-3120176-supplementary.pdf]

Injectable Biodegradable Chitosan–PEG/PEG–dialdehyde Hydrogel for Stem Cell Delivery and Cartilage Regeneration

Xiaojie Lin [1], Ruofan Liu [1], Jacob Beitzel [1], Yang Zhou [1], Chloe Lagadon [1] and Miqin Zhang [1,2,*]

1 Department of Materials Science and Engineering, University of Washington, Seattle, WA 98195, USA; xjlin@uw.edu (X.L.); rfliu@uw.edu (R.L.); jbeitz2@uw.edu (J.B.); yz0426@uw.edu (Y.Z.); clagadon@uw.edu (C.L.)
2 Department of Neurological Surgery, University of Washington, Seattle, WA 98195, USA
* Correspondence: mzhang@uw.edu

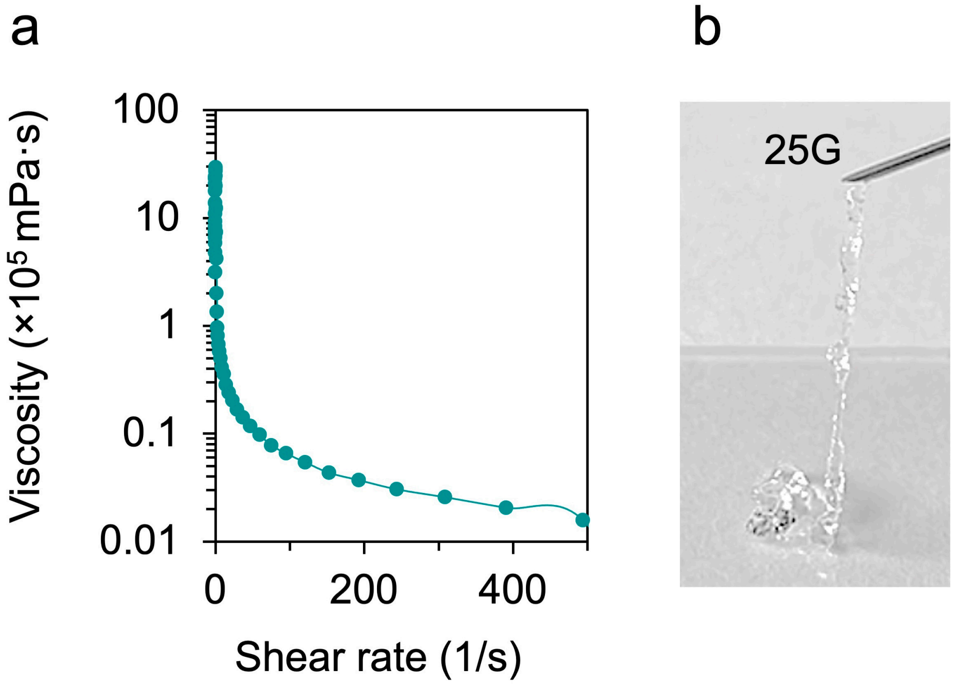

Figure S1. (a) Shear thinning property of chitosan-PEG/PEG-dialdehyde hydrogel, the viscosity of the hydrogel was recorded at a constant shear rate (0.5 s^{-1}) at 37 °C. (b) Illustration of the injection of the hydrogel through a 21 G needle. The hydrogels were prepared by mixing chitosan-PEG (2.5%, w/v, DPBS) and PEG-aldehyde (1g/mL, DPBS) at a volume mixing ratio of 6/1.

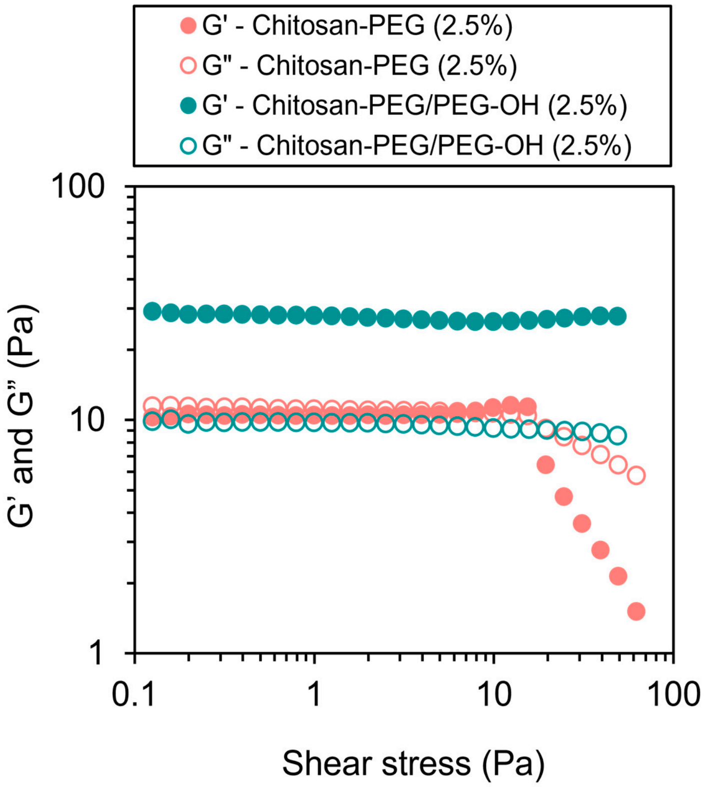

Figure S2. Rheological analysis of chitosan-PEG and chitosan-PEG/PEG-OH mixture. The storage (G', filled circle symbols) and loss moduli (G", empty circle symbols) were recorded under increasing shear stress for uncrosslinked chitosan-PEG and chitosan-PEG/PEG 2000 (PEG-OH) mixture at a concentration of 2.5% (w/v) at 37 °C 1 Hz to estimate the gelation capability and compare their mechanical strength. The current chitosan-PEG showed a comparable storage modulus (G') to loss modulus (G"). The mixture of chitosan-PEG/PEG-OH shows the storage (G') higher than loss moduli (G") independent of the applied stress up to approximately 70 Pa, indicating a gel-like mixture was formed.

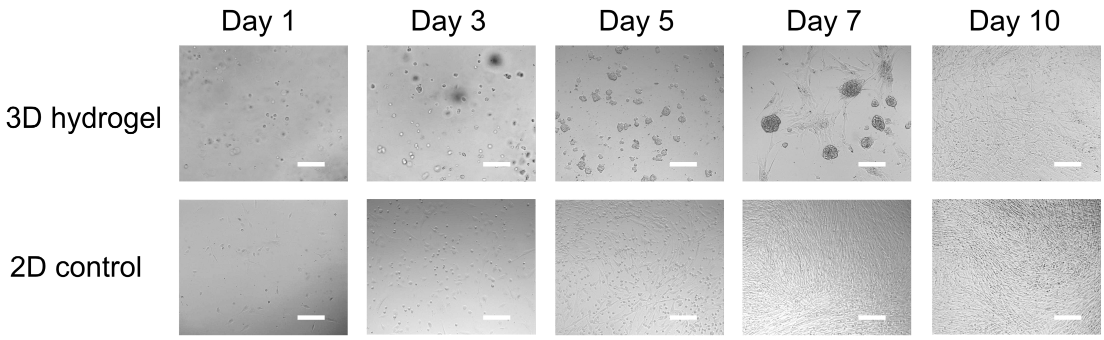

Figure S3. hADSC morphology in hydrogels. The morphology of hADSCs encapsulated in chitosan-PEG/PEG-dialdehyde hydrogels (2.5%, w/v, 3D hydrogel) and on regular 2D surfaces (2D control) on days 1, 3, 5, 7, and 10. Scale bars represent 200 μm.

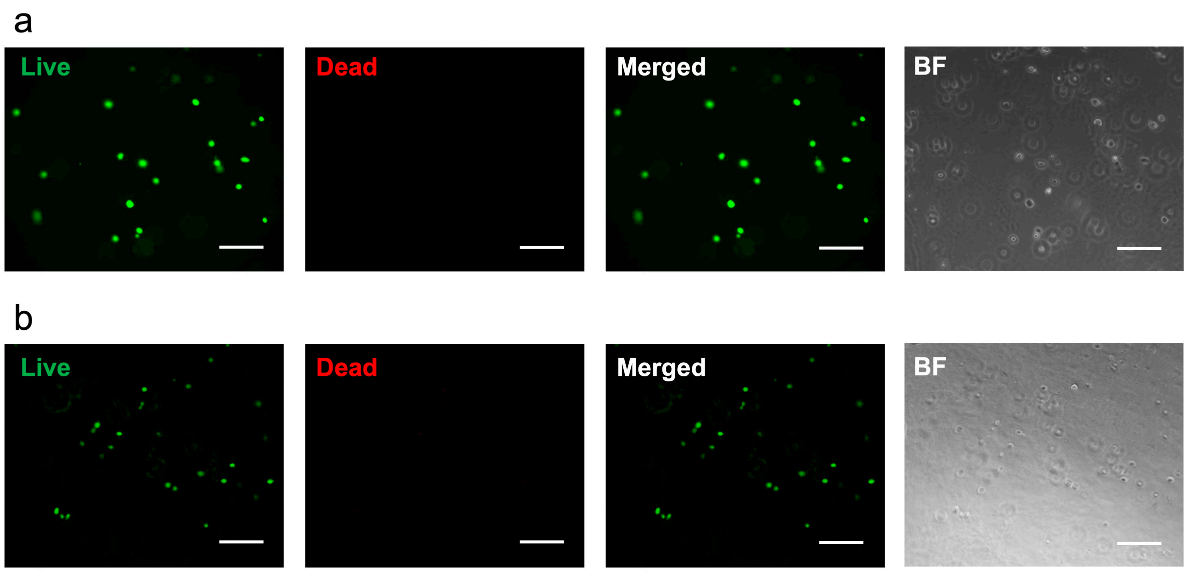

Figure S4. The effect of injection process on hADSCs viability. (a) Fluorescence images of hADSCs encapsulated in chitosan-PEG/PEG-dialdehyde hydrogel. The cell-laden hydrogel was formed in situ by injection of chitosan-PEG/hADSC and PEG-dialdehyde through a double barrel syringe. (b) Fluorescence images of hADSCs encapsulated in chitosan-PEG/PEG-dialdehyde hydrogel. The cell-laden hydrogel was formed by mixing of chitosan-PEG/hADSC and PEG-dialdehyde solution, and subsequently injected through a single barrel syringe. The hADSCs were stained with Calcein-AM (green, for live) and propidium iodide (red, for dead). The scale bars represent 200 μm. No dead cells were found, indicating that the injection will not affect the cell viability.

Table S1. Primer sequences of the reference and target genes.

**Gene**	**Forward**	Reverse
**COL-II**	GTTCACGTACACTGCCCTGA	TCCACACCGAATTCCTGCTC
**ACAN**	AGTCACACCTGAGCAGCATC	TCTGCGTTTGTAGGTGGTGG
**SOX9**	AGGAAGTCGGTGAAGAACGG	AAGTCGATAGGGGGCTGTCT
**GAPDH**	TTGGTATCGTGGAAGGACTCA	TGTCATCATATTTGGCAGGTTT
